# Pig Counting Algorithm Based on Improved YOLOv5n Model with Multiscene and Fewer Number of Parameters

**DOI:** 10.3390/ani13213411

**Published:** 2023-11-03

**Authors:** Yongsheng Wang, Duanli Yang, Hui Chen, Lianzeng Wang, Yuan Gao

**Affiliations:** 1 College of Information Science and Technology, Hebei Agricultural University, Baoding 071001, China; wys981114@163.com (Y.W.); gaoyuan_83_91@126.com (Y.G.); 2 Hebei Key Laboratory of Agricultural Big Data, Baoding 071001, China; 3 College of Animal Science and Technology, Hebei Agricultural University, Baoding 071001, China; 4 Key Laboratory of Broiler and Layer Facilities Engineering, Ministry of Agriculture and Rural Affairs, Baoding 071001, China; 5 Hebei Layer Industry Technology Research Institute, Handan 056007, China; 15030424480@163.com

**Keywords:** pig count, cross scene, FasterNet, YOLOv5n, android application

## Abstract

**Simple Summary:**

Pig counting is important work in the breeding process of large-scale pig farms. Currently, animal counting is mainly observed manually, which leads to increased labor costs and is also prone to animal stress. The noncontact computer vision method avoids the above problems. Therefore, we propose a method for pig counting using machine vision technology. Meanwhile, a pig counting application for the Android system was developed, which truly realized the practical application of the technology.

**Abstract:**

Pig counting is an important work in the breeding process of large-scale pig farms. In order to achieve high-precision pig identification in the conditions of pigs occluding each other, illumination difference, multiscenes, and differences in the number of pigs and the imaging size, and to also reduce the number of parameters of the model, a pig counting algorithm of improved YOLOv5n was proposed. Firstly, a multiscene dataset is created by selecting images from several different pig farms to enhance the generalization performance of the model; secondly, the Backbone of YOLOv5n was replaced by the FasterNet model to reduce the number of parameters and calculations to lay the foundation for the model to be applied to Android system; thirdly, the Neck of YOLOv5n was optimized by using the E-GFPN structure to enhance the feature fusion capability of the model; Finally, Focal EIoU loss function was used to replace the CIoU loss function of YOLOv5n to improve the model’s identification accuracy. The results showed that the AP of the improved model was 97.72%, the number of parameters, the amount of calculation, and the size of the model were reduced by 50.57%, 32.20%, and 47.21% compared with YOLOv5n, and the detection speed reached 75.87 f/s. The improved algorithm has better accuracy and robustness in multiscene and complex pig house environments, which not only ensured the accuracy of the model but also reduced the number of parameters as much as possible. Meanwhile, a pig counting application for the Android system was developed based on the optimized model, which truly realized the practical application of the technology. The improved algorithm and application could be easily extended and applied to the field of livestock and poultry counting, such as cattle, sheep, geese, etc., which has a widely practical value.

## 1. Introduction

The number of pigs keeps changing as a result of elimination and death in farming. The number of pigs needs to be counted during the transfer of pigs, purchasing of feed, and planning of breeding programmers. When auditing agriculture and the selling of pigs, auditors and purchasers also want to obtain the specific numbers of pigs in real time and accurately. Therefore, the study of a pig counting algorithm is of practical significance. Meanwhile, judging the reasonableness of stocking density with counting results is important to ensure the welfare of pig growth as well [1]. At present, pig counting is mainly performed using manual counting. As for the large pig farms, because of the large number of pigs, the counting results of manual counting methods are likely to be inaccurate due to visual fatigue and the influence of subjective factors. At the same time, it is easy to cause stress in pigs with manual counting, which makes pigs frightened and causes them to run around, not only making the counting more difficult but also increasing the risk of zoonotic diseases [2]. The counting method of machine vision does not cause contact with the animal, so it does not cause a stress reaction in the animal. Moreover, the counting method of machine vision is inexpensive, can work for a long time, and can obtain counting results in real time, which has obvious advantages compared with the manual counting method.

Individual recognition is the basis of counting. With the development of information technology, animal identification and counting based on machine vision has become a research hotspot due to the advantages of machine vision technology that does not need to have contact with animals, can be used for a long time, and has a low cost. Early methods of counting using machine vision were mainly implemented with the segmentation of foreground and background [3]. Perner et al. [4] implemented the segmentation of pigs from images by using threshold segmentation and linear overlap methods. Ahrendt et al. [5] achieved pig identification by creating an association between the original map and the corresponding map of individual pigs using a Gaussian model. Buayai et al. [6] achieved the segmentation of pigs by integrating the image adaptive thresholding segmentation method. Using a combination of logarithmic and exponential, background subtraction, and statistical filtering, Poursaberi et al. [7] accomplished the segmentation of individual cows. The literature mentioned above has made progress in individual recognition, but the research methods in the literature require researchers to segment images manually and extract feature parameters manually, so the segmentation effect relies on the researcher’s experience, and the stability of segmentation accuracy cannot be guaranteed.

With the development of deep learning technology, more and more scholars are trying to conduct animal recognition using deep learning technology. Tian et al. [1] implemented the recognition and counting of 21 pigs using a modified ResNeXt structure. Zheng et al. [8] implemented gesture recognition and localization of sows in farrowing pens using the Faster R-CNN model. Using an improved YOLOv4 (You Only Look Once version 4) model, Ahn et al. [9] successfully realized the recognition and counting of 10 pigs. Jensen et al. [10] accomplished the counting of 18 pigs using the technique of Convolutional Neural Networks (CNN) by adding a single linear regression output node. For other animal recognition, Bello et al. [11] achieved the segmentation of a single cow using an improved Mask R-CNN model. Tassinari et al. [12] achieved cow identification with YOLO modeling. Xiao et al. [13] utilized an improved Mask R-CNN model to achieve segmentation of individual dairy cows. Cao et al. [14] implemented sheep identification and counting using an improved YOLOv5x model.

In summary, individual recognition is the basis of counting. Although some research achievements have been made in the research field of pig counting based on deep learning techniques, the factors of the overlapping occlusion between pigs, the undesirable lighting conditions in the barn, and the differences in the size of the pig imaging make it more difficult to identify pigs and result in poor accuracy of counts. Moreover, as for the current deep learning recognition algorithms, the amount of model parameters is too large, which makes it difficult to deploy the algorithms on the mobile Android system and hinders the use and promotion of algorithms in practice. In this paper, a pig counting algorithm based on the improved YOLO v5n was proposed, and at the same time, combined with the Android Studio tool, a pig counting system featuring cross-scenario, high accuracy, and usability in practice was developed. With several datasets of publicly available real scenarios, by using the FasterNet model [15], the improved GFPN structure E-GFPN(Giraffe Feature Pyramid Networks) [16], and the Focal EIoU [17] loss function, the proposed algorithm in this paper was obtained by optimizing the minimal version of YOLOv5, the YOLOv5n model. The algorithm proposed in this paper not only identified pigs in complex environments with high accuracy but also greatly reduced the number of parameters and the number of calculations. The algorithm proposed can be easily generalized and applied to the field of individual identification and the counting of other livestock and poultry, indicating that the method in this paper has great value in the accurate management of livestock and poultry.

The rest of this paper is organized as follows: Section 2 describes the background and related work. Section 3 describes the sources of experimental data and data preprocessing. The improved YOLOv5n model proposed in this paper is introduced in Section 4. Experimental results and discussion are provided in Section 5. Section 6 introduces the steps for deploying the application on the Android platform and how to use the system and demonstrates the recognition effect. The conclusion with future research directions is shown in Section 7.

## 2. Related Work

Automated and accurate livestock counting plays a key role in the intelligent management of factory livestock farming. Computer vision perception is the main technology currently used for livestock counting. Early visual pig counting methods achieved pig counts by segmenting the foreground from the background using a combination of morphological algorithms and area growth methods [18,19]. However, these methods need to extract the feature parameters of the image manually. It is difficult to extract the feature parameters, and the model has poor generalization and robustness.

The rise in deep convolutional neural networks provides a new idea for pig counting. Deep convolutional neural networks could extract data features by learning autonomously from a large number of samples and have excellent performance in target recognition. More and more scholars are trying to conduct animal counting with deep learning. Density map regression counting methods based on neural networks have been favored by a number of researchers [20,21]. These methods convert the image into a density map regression by using pixels and integrating the density map to obtain the counting results. Chen et al. [22] proposed a bottom-up method for detecting the critical points of the pig’s body and then performing pig counting based on the STRF (Spatially Sensitive Timing Response) method. Hu et al. [23] solved the problem of missing pig targets due to light and pig occlusion using a modified Mask R-CNN network, and the accuracy of counting 12 to 22 pigs in a single cage reached 98%. Yang et al. [3] improved the YOLOv5n model by introducing the SE Net channel attention module and solved the problem of misdetection and missed detection due to occlusion, changes in body size, and changes in light by using the improved model, and the counting accuracy reached 99.39%.

However, the high accuracy of these methods is based on deeper, broader, and more complex network structures. Along with the performance of the network increasing, the complexity of the network model and the number of parameters also increase dramatically. Complex models demand very high performance of the device, so it cannot be directly deployed in embedded systems, limiting the application of deep learning techniques in livestock and poultry production. To realize the practicality of the model, the FasterNet model was used to replace the Backbone part of YOLOv5n, reducing the number of parameters as far as possible while maintaining the performance. Requirements for the performance of the equipment are reduced, providing the possibility of online, real-time deployment of the model.

## 3. Materials and Methods

### 3.1. Data Sources and Analysis

**Figure 1:**
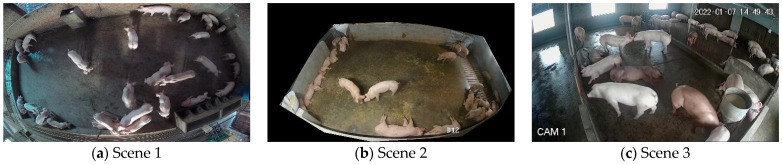
Examples of pig images in different scenarios.

The experimental data were mainly collected from the “Pig Counting Challenge” [24] on the iFlytek platform and the pig target detection dataset provided by Alibaba Cloud Tianchi Lab. The “Pig Counting Challenge” provides 700 training set images and 220 test set images. The training set image resolution is 1536 × 2048 pixels and 1920 × 1080 pixels, the test set image resolution is 1920 × 1080 pixels, the data of the training set and the test set were collected from different pig houses, and the 881 images with better quality were selected. From the pig target detection dataset provided by AliCloud Tianchi Lab, 51 images with good quality (image resolution of 1920 × 1080 pixels) were chosen. The 51 images selected from Alibaba Cloud Tianchi Lab and the 881 images selected from the “Pig Counting Challenge” constitute the final primary dataset. The dataset includes a total of 932 images in 3 different scenes. Some of the pig images from the 3 different scenes are shown in Figure 1.

### 3.2. Data Preprocessing and Dataset Construction

**Figure 2:**
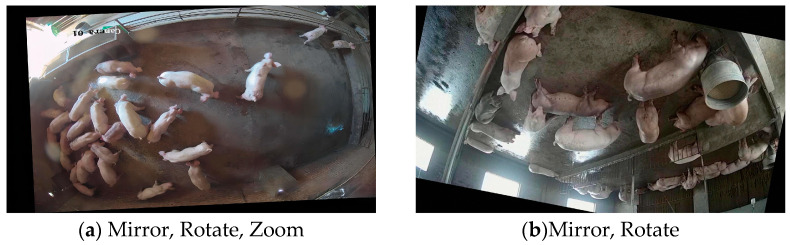
Data enhancement effect.

After the dataset collection was conducted, firstly, the data were cleaned in order to remove the similar images and ensure the difference between the images. After data collection was conducted, firstly, the data were cleaned to remove similar images and to ensure the difference between the images. Then, the original dataset was labeled using the Labelling tool to generate a pig identification dataset satisfying the Pascal VOC [25] format. Subsequently, the training, validation, and test sets were generated from the dataset by selecting images randomly, in the ratio of 6:2:2, using a scripting program written with Python language. In order to enhance the robustness of the algorithm [26], the images in the training set have been enhanced offline by using randomly selected rotation, scaling, and mirroring. The enhanced result is shown in Figure 2.

**Figure 3:**
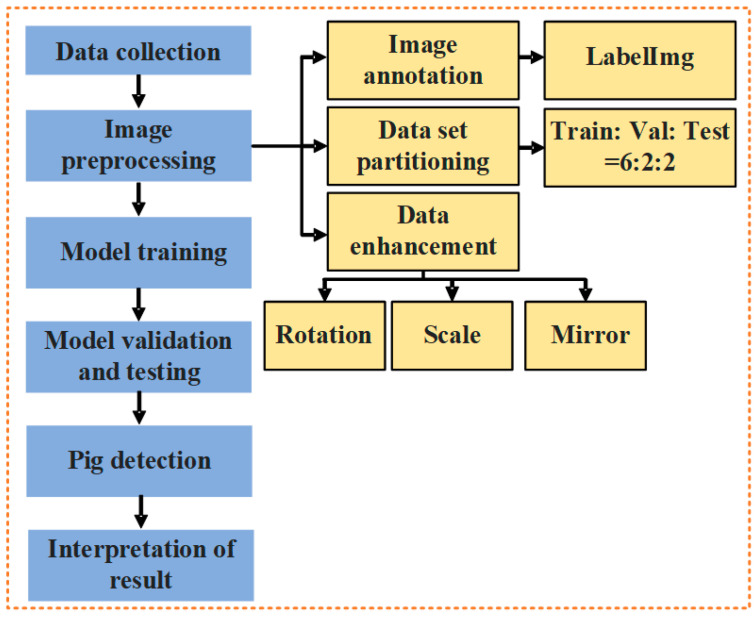
General flow chart of the experiment.

After preprocessing, the recognition dataset has a total of 2609 images, of which the training, validation, and test sets are 2236, 186, and 187. The relevant data from the final pig count dataset are shown in Table 1. The dataset construction process is shown in Figure 3.

As shown in Figure 3, firstly, different scene images were collected to produce a cross-scene pig counting dataset; secondly, images were preprocessed to prepare for model training; thirdly, testing performance and analyzing results were conducted after training of the model; finally, the model was deployed on the Android system.

## 4. Pig inventory Methods

### 4.1. YOLOv5n Network Structure

**Figure 4:**
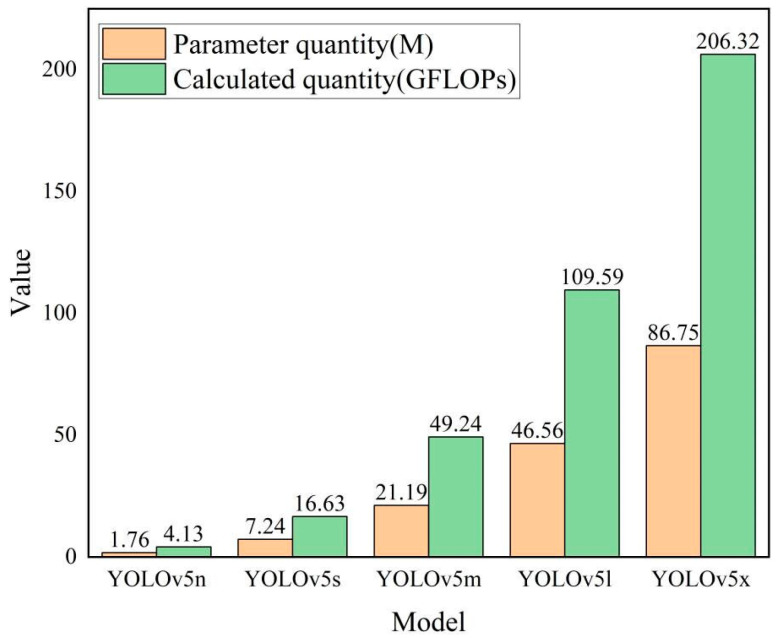
YOLOv5 parametric quantities and operations by version.

The inference speed and detection accuracy of the YOLOv5 model [27] are significantly improved over that of the YOLOv4 model. According to the depth and width of the network structure, the YOLOv5 model can be classified into five versions, namely YOLOv5n, YOLOv5s, YOLOv5m, YOLOv5l, and YOLOv5x, of which the YOLOv5n model is the smallest version. A comparison of the number of parameters and calculations for each version is shown in Figure 4.

**Figure 5:**
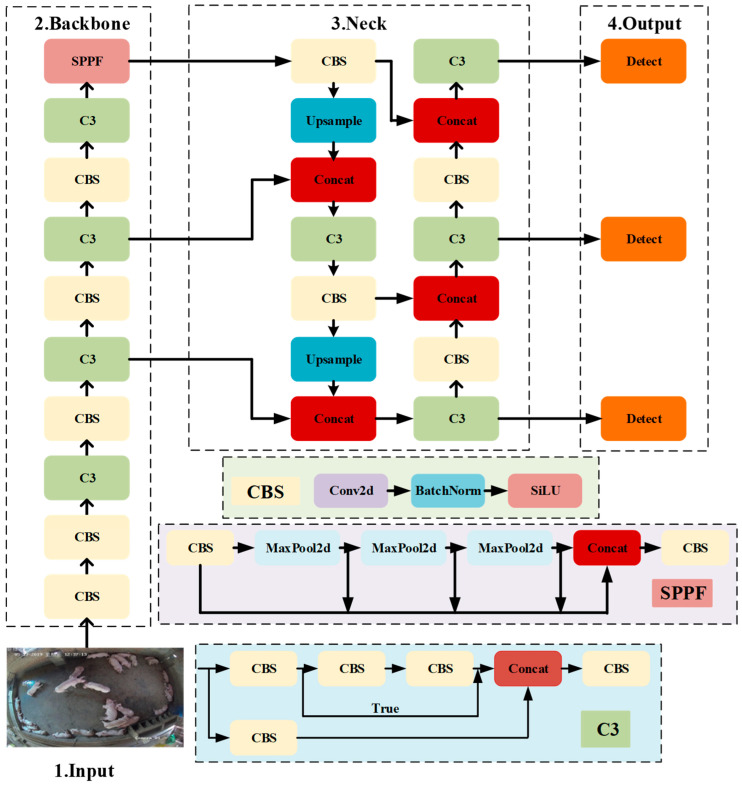
YOLOv5n model structure diagram.

To be easily deployed on the Android system, the YOLOv5n was chosen as the base model for pig counting. The structure of the YOLOv5n model is shown in Figure 5.

There are four parts of the YOLOv5n model: the Input, the Backbone, the Neck, and the Output. The Input part is used to input preprocessed pig images to the model; the Backbone part mainly employs the C3 module with CSP structure for extracting the semantic information of the target; the Neck part uses a PANet (Path Aggregation Network) structure, which first extracts features from each layer of the network with a top-down FPN (Feature Pyramid Network) structure and then learns the underlying positional information using a bottom-up path so that the feature fusion ability on multiple scales can be enhanced [28]. The Output part consists of three detection heads with different feature map sizes, which are used to recognize small, medium, and large targets, respectively, and output the recognition results.

### 4.2. Improved YOLOv5n Network

The CSP structure in the YOLOv5n divides the original input into two branches, and the two branches perform the convolution operation separately, thereby increasing the number of parameters of the model. As a result, the model is not easy to be deployed in mobile terminals [29]. The occlusion of pigs from each other, smaller imaging of pigs far away from the camera, and strong or poor lighting could reduce the effective characteristics of pigs, resulting in missed or multiple counts using the model. In order to solve the problem of missed or multiple counts, a counting method based on the improved YOLOv5n model is proposed. The detailed procedure of the method is as follows:

1. Using the FasterNet model as the Backbone of the YOLOv5n;
2. The neck of YOLOv5n is optimized using an improved GFPN structure E-GFPN;
3. Replacing the CIoU loss function of the YOLOv5n with the Focal EIoU loss function.

**Figure 6:**
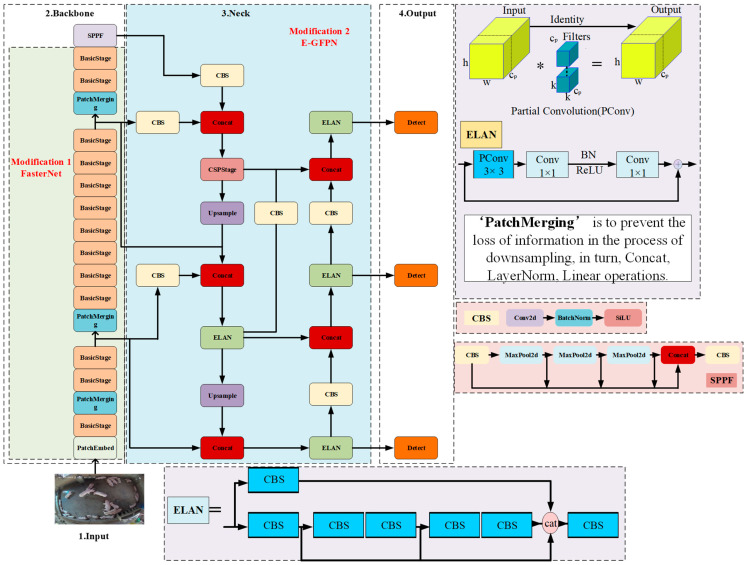
Improved YOLOv5n model structure diagram. * Conv.

The structure of the improved YOLOv5n model is shown in Figure 6.

#### 4.2.1. Improvement of Backbone Network

To reduce the number of parameters and calculations so that the model can be easily deployed on portable and mobile devices and to enhance the practical value of the pig counting algorithm, the FasterNet model was used to replace the Backbone part of the YOLOv5n.

**Figure 7:**
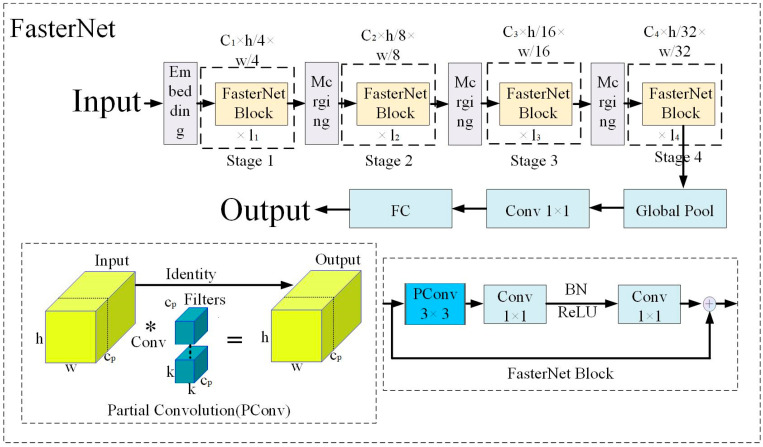
FasterNet model structure diagram.

The FasterNet model enhances the processing speed by utilizing the Partial Conv operation (PConv). The PConv operation avails the redundancy of feature maps and performs regular convolution (Conv) on only a part of the input channels. There is no impact on the remaining channels, which not only reduces redundant computations but also reduces memory access [15]. The structure of FasterNet is shown in Figure 7.

As seen in Figure 7, the FasterNet model has four hierarchical levels. Every hierarchical level is preceded by an embedding layer (regular 4 × 4 convolution with a step size of 4) or a merging layer (regular 2 × 2 convolution with a step size of 2) for spatial downsampling and number of channel expansion. After every hierarchical level, there are several FasterNet Blocks. The FasterNet Block has a PConv layer followed by 2 PWConv (or Conv 1 × 1) layers, the middle layer has an extended number of channels, and the FasterNet Block uses Shortcut connections to reuse input features.

#### 4.2.2. Improvement of Neck Section

The Neck of the YOLOv5n fuses the 3 different scales of feature maps coming from the Backbone part. When the input image size is 640 × 640 pixels, the scales of the three feature maps are 20 × 20 pixels, 40 × 40 pixels, and 80 × 80 pixels. Feature maps of 3 different scales with decreasing receptive fields were used to fuse large, medium, and small targets. By using the Neck of the YOLOv5n for feature fusion, since the scales of the three feature maps are fixed, the feature map with the largest receptive field may also fail to extract effective high-level semantic information for some super-large scale pig images, and the feature map with the smallest receptive field may also lose the pig’s outline and location information for some super-small scale pig images [3]. In the actual breeding environment, due to the different distances from the camera, there will be pigs that are close to the camera and have a super-large scale, and there will also be pigs that are far from the camera and have a super-small scale. Moreover, the mutual occlusion between pigs and the different lighting of the pig house will make the effective features of some pigs insufficient or lost, so it is difficult to achieve a good fusion effect by directly utilizing the Neck part of the YOLOv5n model.

**Figure 8:**
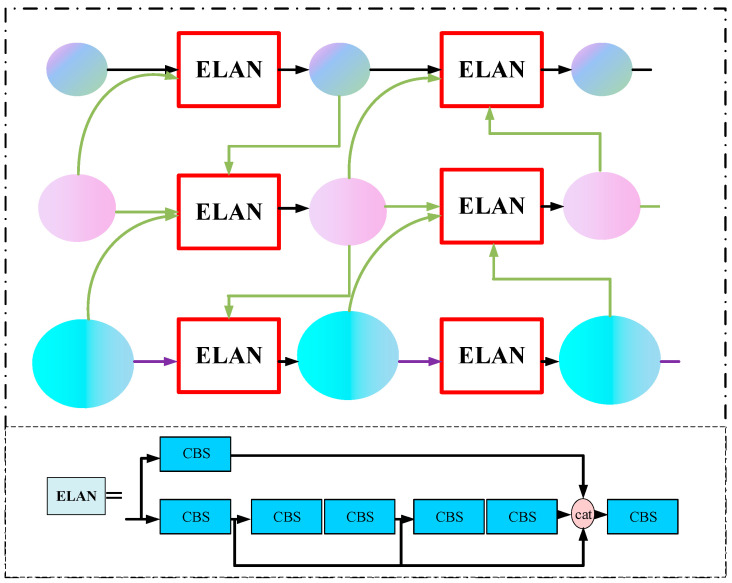
E-GFPN structure diagram.

The GFPN structure not only integrates different scale feature information in a top-down and bottom-up manner but also fuses internal feature information, enhancing the model’s ability to interact with spatial information in high-resolution feature maps and semantic information in low-resolution feature maps. The ELAN module controls the shortest and longest gradient paths of the model, further enhancing its learning capacity. The Fusion Block module in the GFPN structure is replaced with the ELAN module, forming the E-GFPN structure, which can further enhance the feature fusion capability of the GFPN structure. Therefore, to address the problem of insufficient, lost, and missed effective features due to occlusion and distant imaging of pigs away from the camera end, this study utilizes the E-GFPN structure to replace the PANet structure at the Neck end of the YOLOv5n model, and the fusion ability of the model for effective pig features is enhanced by fusing features of different scales. The E-GFPN structure is shown in Figure 8.

#### 4.2.3. Improvement of Loss Function

**Figure 9:**
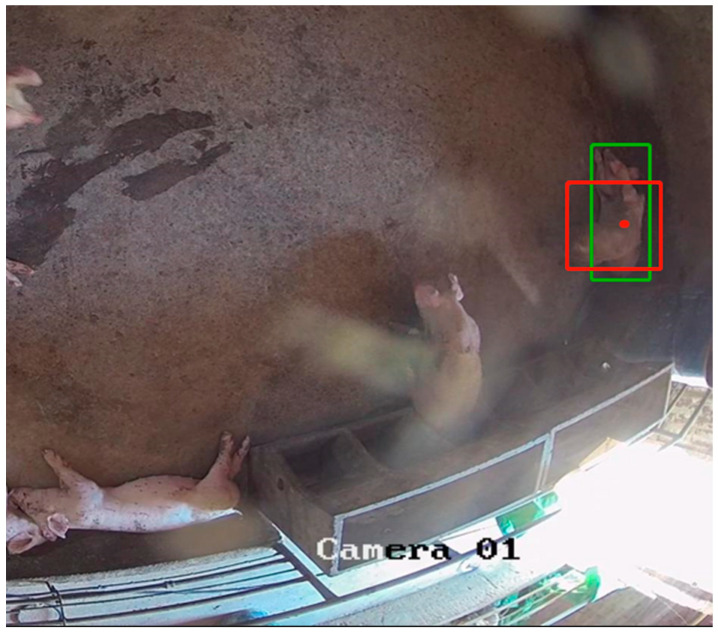
Schematic diagram of the same center point of the two targets.

Although the CIoU loss function in the YOLOv5n model takes into account the ratio of the width and height of the Bounding Box and the differential distance between the center of the real box and the predicted box, the CIoU loss function only uses the ratio of the width and height as an influencing factor for the computation of the loss of the bounding box. When the center point of the two boxes is the same as that of the original graph (as shown in Figure 9, red and green indicate the target boxes of different pigs, respectively), the ratio of the width and height of the two boxes is the same, but the values of the width and height are different, which leads to the deviation between the regression results and the real values.

Mutual occlusion between pigs causes the effective features of the occluded section to be missing, resulting in missed detections or misdetection by the model. To enhance the detection accuracy, the CIoU loss function of YOLOv5n was replaced by *Focal EIoU* [17].

*Focal EIoU* is based on the *EIoU* loss function by introducing Focal Loss to focus on quality anchor frames, which not only has better localization of the target in the regression process but also solves the imbalance problem of difficult and easy samples. The *EIoU* loss function consists of 3 parts, which are *IoU* loss, distance loss, and width–height loss. The height–width loss directly minimizes the difference between the height and width of the predicted target bounding box and the real bounding box, enabling *EIoU* loss functions to localize better. Therefore, using the *Focal EIoU* loss function could better localize the occluded region and solve the problem of balance between difficult and easy samples, which could improve the pig counting accuracy effectively. The formula of the *EIoU* loss function is shown in Equation (1).

(1) $$L_{EIoU}=L_{IoU}+L_{\mathrm{dis}}+L_{\mathrm{asp}}=1-IOU+\frac{\rho^{2}(b,b^{{}^{gt}})}{{\left(\omega^{c}\right)}^{2}+{\left(h^{c}\right)}^{2}}+\frac{\rho^{2}(\omega ,\omega^{{}^{gt}})}{{\left(\omega^{c}\right)}^{2}}+\frac{\rho^{2}(h,h^{{}^{gt}})}{{\left(h^{c}\right)}^{2}}$$

where *ω^c^* and *h^c^* are the width and height of the minimum outer rectangle of the predicted and real bounding boxes, respectively, and *ρ* is the Euclidean distance between the two points.

*Focal EIoU* loss function combines Focal Loss loss function based on *EIoU* loss function. The *Focal EIoU* loss function separates high- and low-quality anchor frames and solves the imbalance between hard and easy samples from the perspective of the gradient. The calculation of the *Focal EIoU* loss function is shown in Equation (2).

(2) $$L_{Focal-EIoU}=IOU^{\gamma }L_{EIoU}$$

### 4.3. Experimental Configuration

The hardware configuration used in this study is as follows: the CPU model is Intel Core i7-9700, and the GPU model is Nvidia GeForce RTX3060, with a VRAM capacity of 12 GB. The programming language Python 3.8, Pytorch 1.9 Deep Learning Framework, and CUDA 11.3.58 Deep Learning Acceleration Library are installed on the Windows 10 operating system. The hyperparameter settings used are shown in Table 2.

### 4.4. Performance Evaluation

Precision (P), Recall (R), Average Precision (AP), and F1-Score are used for the evaluation of model recognition accuracy. Model complexity is evaluated using the number of parameters, calculations, and model size. The FPS is used to evaluate the speed of model detection.

Precision represents the ratio of correctly detected pig targets to all pig targets identified, Recall denotes the ratio of the number of pigs that were detected as a positive category, and the true category was also a positive category to the total number of all pigs whose true category was positive. F1-Score denotes the reconciled average of Precision and Recall, and AP denotes the results of the detection of all categories (in this experiment, there was only one category of pigs). The Equations are shown in (3)–(6):

(3) $$P=\frac{TP}{TP+FP}\times 100\%$$

where *TP* is the number of sample of pigs predicted accurately by the model and *FP* is the number of sample of pigs mispredicted by the model.

(4) $$R=\frac{TP}{TP+FN}\times 100\%$$

where *FN* is the number of samples of pigs missed by the model.

(5) $$AP=\int_{0}^{1}P(R)dR$$

(6) $$F1=\frac{2}{\frac{1}{P}+\frac{1}{R}}=\frac{TP}{TP+\frac{\left(FP+FN\right)}{2}}$$

The model’s counting performance is evaluated by using mean absolute error (MAE) and root mean square error (RMSE).

*MAE* denotes the difference between the prediction value and the true value, and *RMSE* denotes the average absolute difference between each predicted value and the Y = X line in the scatterplot. The Equations are shown in (7) and (8):

(7) $$RMSE=\sqrt{\frac{\sum_{i=1}^{n}{\left(predicted_{i}-actual_{i}\right)}^{2}}{n}}$$

(8) $$MAE=\frac{\sum_{i=1}^{n}\left|predicted_{i}-actual_{i}\right|}{n}$$

where *n* is the number of true or predicted values, *predicted_i_* is the *_i_*th predicted value, and *actual_i_* is the *_i_*th true value.

## 5. Results and Analysis

To the best of our knowledge, our algorithm is the first one to minimize the number of parameters while maintaining accuracy. Overall, the contributions of our paper can be summarized in three parts:

- Collected image data from several different pig farms and produced a pig counting dataset to enhance the generalization of the model.
- Used the FasterNet model as the Backbone part of YOLOv5n; the number of parameters, the calculations, and the size of the model were reduced by 50.57%, 32.20%, and 47.21% compared to YOLOv5n, and the detection speed reached 75.87 f/s.
- Combined with Android Studio tools, we developed a cross-scenario, high-precision, and practical pig counting system, which was convenient for farmers to know the number of pigs anytime, anywhere, and on time.

### 5.1. Performance Evaluation

**Figure 10:**
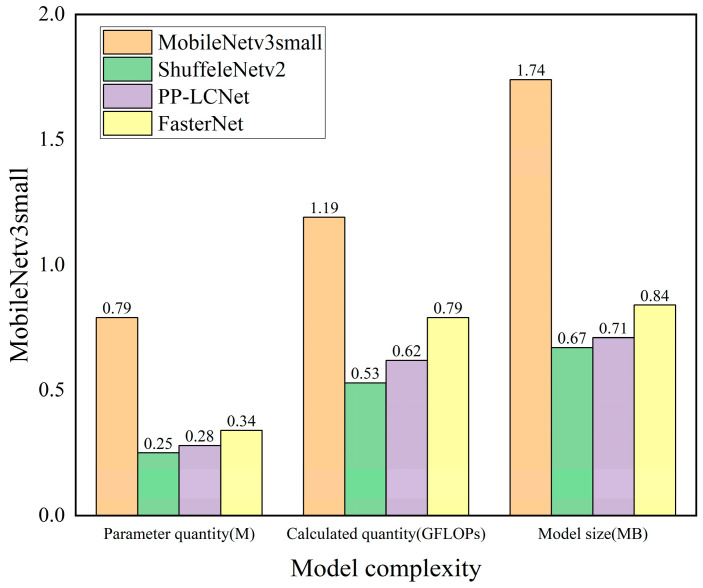
Model complexity comparison chart.

We replaced the Backbone part of the YOLOv5n model with four currently mainstream lightweight models, MobileNetv3s-mall [30], ShuffleNetv2 [31], PP-LCNet [32], and FasterNet [15], respectively, to improve the YOLOv5n model. Using the dataset constructed in this paper, the four improved models were trained, and the det30ection accuracy of the four improved models is shown in Table 3. Comparisons of model complexity are shown in Figure 10.

From Table 3, it is found that in terms of model detection accuracy, the Precision, Recall, F1-Score, and AP of the model replaced by FasterNet are 94.72%, 89.42%, 91.99%, and 95.64%, respectively. Although the Precision is slightly lower than that of the model replaced by ShuffleNetv2 and PP-LCNet, the Recall, F1-Score, and AP are higher than those of the other models. The AP is 2.30, 1.99, and 1.36 percentage points higher than that of the models replaced by MobileNetv3small, ShuffleNetv2, and PP-LCNet, respectively.

A comparison of Figure 10 reveals that in terms of model complexity, the number of model parameters, the number of calculations, and the model size replaced by FasterNet are 0.34 M, 0.79 GFLOPs, and 0.84 MB, respectively. The indicators are almost the same as those of models replaced by ShuffleNetv2 and PP-LCNet and are significantly lower than those of models replaced by MobileNetv3small.

Comprehensively considered, the performance of the model improved with FasterNet is optimal.

### 5.2. Ablation Experiments

With the ablation test, combining the AP, number of parameters, amount of calculation, and size of the model, the recognition effect of the improved method is evaluated. The experimental results are shown in Table 4. Table ‘A’ denotes the YOLOv5n model. ‘B’ is the model with the Backbone of YOLOv5n replaced by FasterNet. ‘C’ is the model with Backbone replaced by FasterNet and Neck replaced by E-GFPN of YOLOv5n. ’D’ is the model with Backbone replaced by FasterNet and Neck replaced by E-GFPN, and Focal EIoU loss function is used.

From Table 4, it is found that although the AP of model B is 1.91 percentage points lower than that of A, the number of parameters, the number of calculations, and the model size of B are only 19.32%, 19.13%, and 23.46% of that of A. Compared to A, the complexity of B is significantly reduced, which shows that it is effective in reducing the model complexity by replacing Backbone with the FasterNet model. Model C is generated by adding the E-GFPN structure to model B. The complexity of C is slightly increased compared to model B, but there is still a significant decrease in complexity compared to the original model A. Moreover, the AP of model C is significantly improved compared to that of model B, which indicates that the improved E-GFPN structure plays a promotion role in recognition. Based on model C, the loss function Focal EIoU is introduced, and the complexity of the model is unchanged compared to model C, but the AP is improved by 0.23 percentage points, indicating that the improved loss function can further improve the model recognition accuracy. Model D improves AP by 0.17 percentage points in terms of model detection accuracy compared to the original Model A; in terms of model complexity, the number of parameters, the amount of calculation, and the size of model D are only 49.43%, 67.80%, and 52.79% of the original model A. The model complexity has been significantly reduced. Therefore, the optimization model proposed in this paper is more suitable for mobile applications than the YOLOv5n model.

**Figure 11:**
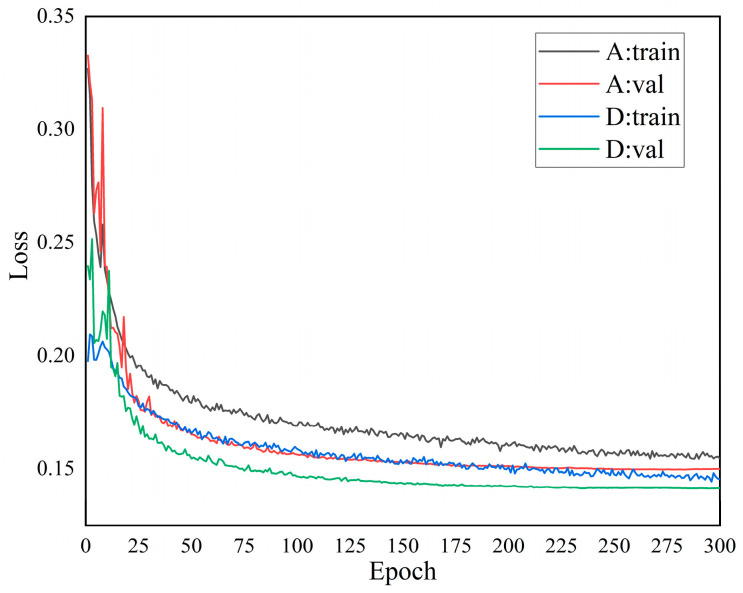
Comparison of model loss value change curves.

The variation curves of the total loss during the training and validation of the YOLOv5n model and the proposed model are shown in Figure 11.

As can be seen in Figure 11, the model loss value decreases quickly at the beginning of the training or validation phase. When trained to 75 Epochs, the loss values were still decreasing, but the trend slowed down. When trained to 300 Epochs, the loss curve flattened out. Compared to the YOLOv5n model, the total loss values of training and validation of the model in this paper are smaller, and the fit is better.

### 5.3. Different Deep Learning Detection Algorithms

To further validate the performance of the proposed algorithm in this paper, the model in this paper is compared with YOLOv3-tiny [33], YOLOv4-tiny [34], YOLOv6n [35], YOLOv7-tiny [36], and YOLOv5n [37] for recognition. Using the pig counting dataset constructed in this paper, the recognition accuracy and complexity of the model are evaluated using Precision, Recall, F1-Score, AP, number of parameters, number of calculations, and model size. The speed of model detection is evaluated with FPS. The recognition results are shown in Table 5, and the model performance comparison results are demonstrated in Table 6.

As can be seen from Table 5, in terms of model detection precision, the mean values of Precision, Recall, F1-Score, and AP of the models proposed in this paper are 97.45%, 93.17%, 95.26%, and 97.72, which are almost the same as the YOLOv5n, YOLOv6n, and YOLOv7-tiny models, and higher than the YOLOv3-tiny model by 3.13, 8.58, 6.07, and 4.79 percentage points, and increased by 2.44, 5.60, 4.12, and 3.12 percentage points over the YOLOv4-tiny model. In terms of detection accuracy, the model proposed can meet the detection task.

From Table 6, it can be seen that in terms of complexity, the number of parameters of the proposed model is 0.87 M, the number of calculations is 2.80 GFLOPs, and the model size is 1.89 MB. The three evaluation indicators are significantly better than those of YOLOv3-tiny, YOLOv6n, and YOLOv7-tiny models. There is a reduction of 2.19 M, 3.5 GFLOPs, and 4.03 MB compared to the YOLOv4-tiny model 0.89 M, 1.33 GFLOPs, and 1.69 MB, which is lower than that of the YOLOv5n model. It is shown that in terms of model complexity, the model proposed in this paper has a significant advantage.

In terms of detection speed, the FPS of the proposed model is 75.87 f/s, which is significantly higher than that of the YOLOv6n and YOLOv7-tiny models and 4.34 f/s higher than the YOLOv5n model. It is shown that the proposed model in this paper has some advantages in the detection speed.

To summarize, considering the three factors of detection accuracy, complexity, and detection speed, the model proposed has obvious advantages in recognition and practical application.

### 5.4. Comparison with Existing Methods

To verify whether the model in this paper has an advantage over other models, the model in this paper is compared with the models in Tian et al. [1], Yang et al. [3], Ahn et al. [9], and Hu et al. [23]. The results of the comparison are shown in Table 7.

From Table 7, it can be seen that in terms of the number of model parameters, the number of parameters of the proposed model is only 27.88%, 1.64%, and 1.01% of the model Yang et al. [3], Ahn et al. [9], and Hu et al. [23], which is significantly lower. Regarding the speed of detection, the model results in this paper are 52.06, 58.01, 42.36, and 73.37 higher than those in Tian et al. [1], Yang et al. [3], Ahn et al. [9], and Hu et al. [23], which is a significant advantage. For the detection time of a single image, the model in this paper spends the least time and costs only 30.95%, 23.21%, 43.33%, and 3.25% of the models of Tian et al. [1], Yang et al. [3], Ahn et al. [9], and Hu et al. [23], which is also a big advantage. In summary, compared with current algorithms for pig counting, the model in this paper has significant advantages in terms of the speed of detection and the deployment on mobile.

### 5.5. Analysis of Test Results

#### 5.5.1. Analysis of Recognition Effect under Different Occlusion Situations

**Figure 12:**
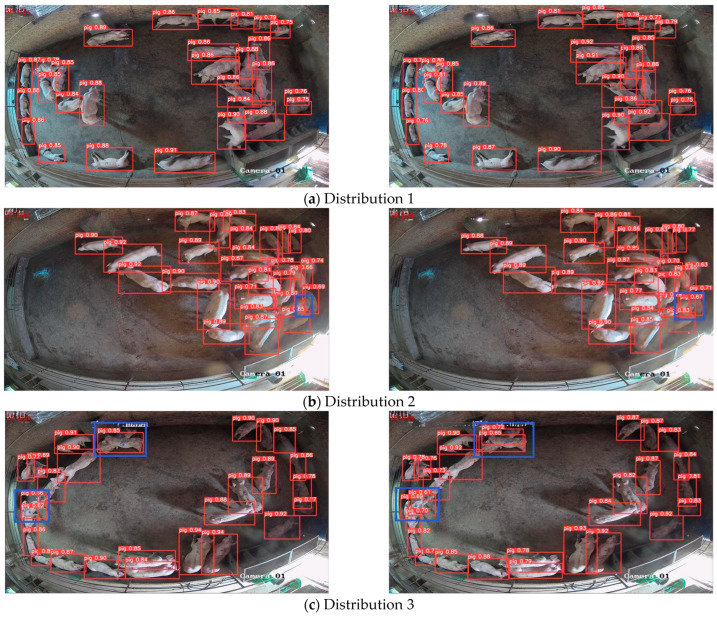

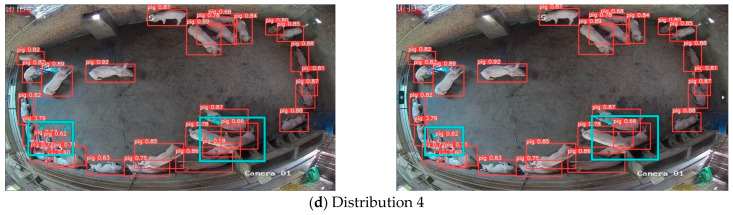
Model recognition renderings under different distribution conditions.

Occlusion is one of the main factors affecting the accuracy of pig identification. To verify that the model proposed has a good recognition effect in different occlusion situations, four images with different distributions in the same scene are randomly selected from the test set and recognized using the YOLOv5n model and the model proposed. The recognition effects are shown in Figure 12. The left Figure shows the recognition effect of YOLOv5n, and the right Figure shows the recognition effect of the proposed model in this paper.

Figure 12a shows the recognition effect of the YOLOv5n and the model proposed in the case that pigs are widely distributed throughout the house, with less mutual occlusion. As can be seen in the Figure, pigs were identified accurately with both models. Figure 12b–d demonstrates the recognition effect of the two models under the conditions of pigs’ aggregation, severe occlusion among individuals, and missing information on pig contour. From Figure 12b,c, it can be seen that missed detection occurs with the YOLOv5n model (as marked by the blue box in Figure). From Figure 12d, it can be seen that a misdetection occurred and that one pig was detected as two with the YOLOv5n model (as marked by the green box and arrow in Figure), while the improved model could recognize it accurately.

#### 5.5.2. Analysis of Recognition Effect under Different Light Conditions

**Figure 13:**
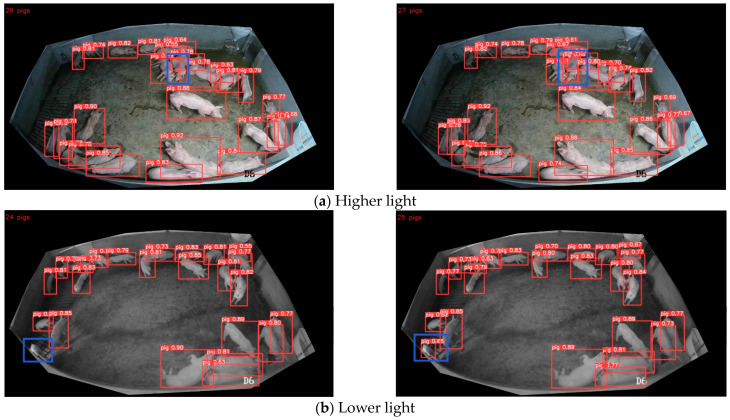
Model recognition renderings under different lighting.

In real environments, light affects accuracy as well. Two images with obviously different lighting are selected randomly from the test set and recognized using YOLOv5n and the proposed model. By comparison, the identification effect in different lighting of the proposed model is verified. The effect is shown in Figure 13; the left Figure is the effect of YOLOv5n, and the right Figure is the effect of the model in this paper.

At the location marked by the blue box in Figure 13a, the light is strong, and the YOLOv5n model (left picture) misses the pig, while the model in this paper (right picture) identifies the pig accurately. At the location marked by the blue box in Figure 13b, the light is weak, the pig outline is unclear, and the YOLOv5n model (left picture) missed the pig. No misses or misdetections occurred with the model in this paper (right picture). The above examples show that the model in this paper has strong robustness.

#### 5.5.3. Analysis of Recognition Effect in Different Scenes

**Figure 14:**
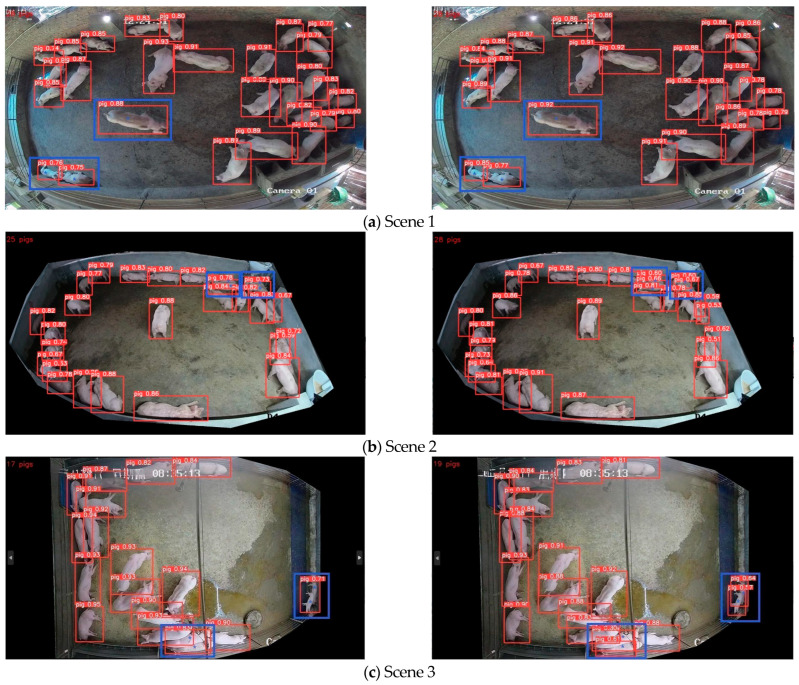
Renderings of pig identification in different scenarios.

Different numbers of pigs and different imaging sizes in different scenarios affect the recognition performance of the model. Three images were selected randomly from the test set to verify the recognition effect of the model proposed by comparison. The effect is shown in Figure 14; the left Figure shows the effect of YOLOv5n, and the right Figure shows the effect of the model in this paper.

Observation from Figure 14 finds that in scene 1, although each pig was recognized by both the unimproved model and the improved model, the confidence thresholds of the pigs recognized by YOLOv5n were lower (as shown at the location marked by the blue box in Figure 14a, left picture). In Scenes 2 and 3, because of the smaller pig imaging away from the camera and the pigs sticking closely together (as shown at the location marked by the blue box in Figure 14b,c), which resulted in fewer effective features, a missed detection occurred in the YOLOv5n model (left picture), whereas the model in this paper (right picture) was able to recognize each pig accurately. It is shown that the model in this paper has better performance and more practicality in recognizing pigs in different scenarios.

### 5.6. Analysis of Counting Errors of Different Models

The counting effect of the proposed model is verified using the mean absolute error and root mean square error by comparing the counting results of YOLOv3-tiny, YOLOv4-tiny, YOLOv6n, YOLOv7-tiny, YOLOv5n, and the model in this paper. The results are shown in Table 8.

As seen from Table 8, in the process of pig counting, the average absolute error of the counts of the model in this paper is 1.37, and the root mean square error is 2.10; the two indicators are slightly higher than that of the YOLOv7-tiny model, but both are better than that of the YOLOv3-tiny, YOLOv4-tiny, YOLO 5n, and YOLOv6n models. Compared to the YOLOv3-tiny model, the mean absolute error and root mean square error were reduced by 1.71 and 2.21. The mean absolute error and root mean square error were reduced by 1.40 and 2.00 compared to the YOLOv4-tiny model. Compared to the YOLOv5n and YOLOv6n models, the mean absolute error and root mean square error were reduced by 0.09 and 0.08. The comparison shows that the model in this paper is competent for pig counting.

## 6. Deployment and Application on Android Devices

The results of pig counting can provide a basis for accurate feeding and asset evaluation. For the convenience of farmers to know the number of pigs anytime, anywhere, and in time, the model proposed in this paper was deployed on an Android system.

### 6.1. Steps for Deploying on Android Devices

**Figure 15:**
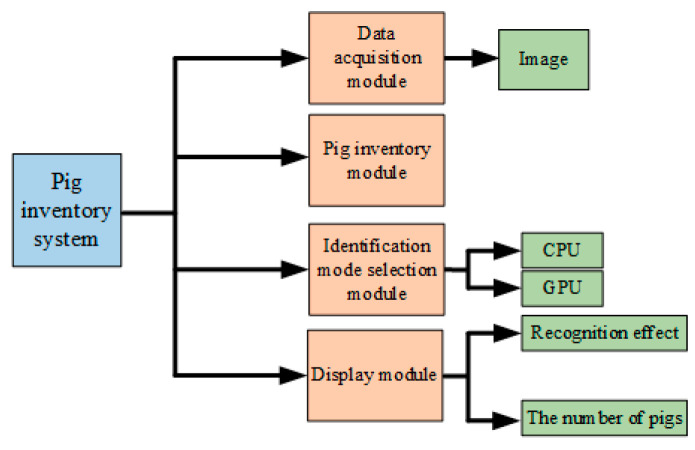
The overall frame diagram of the Android terminal application.

The best.pt weight file trained using the model in this paper was converted into an ONNX format file, and then the ONNX file was compiled into bin and param format files using the NCNN platform. The compiled file was modified using the Netron visualization tool to remove the slicing operations from the original model file. Finally, the modified model file was compiled using the Android Studio tool and written to the Android system to generate the application. The general framework of the Pig Count Android application is shown in Figure 15.

The data acquisition module is used to select an image for pig counting. The pig counting module identifies the images transmitted from the data acquisition section using the trained weights file. The recognition mode selection module provides the selection of CPU or GPU, which is convenient for users to choose according to their terminal configuration. The display module is used to demonstrate the results of the pig counting and the effects of the identification.

### 6.2. Demonstration of System Usage and Effectiveness

**Figure 16:**
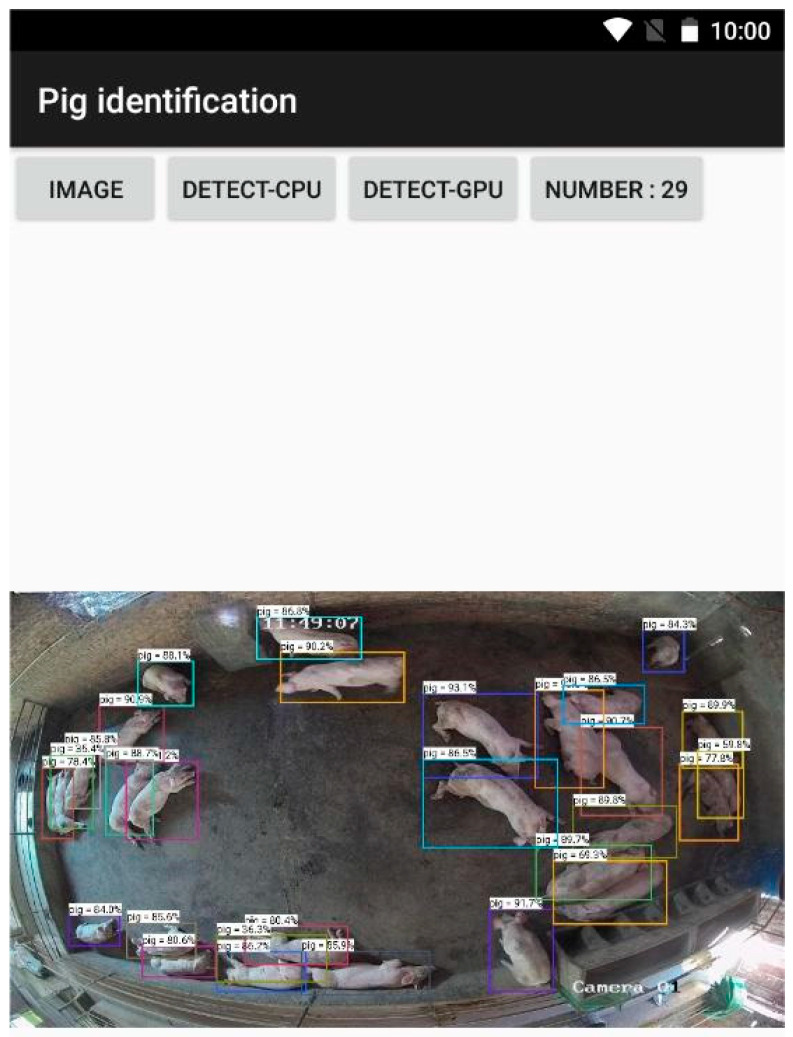
Test effect diagram.

The Huawei Honor v10 was used in this article as the Android test device. The GPU of the device was Kirin 970, running RAM was 4 GB, storage was 64 GB, and the operating system was Android 8.0. The recognition effect on the Android terminal is shown in Figure 16. The user opens the Android app, enters the pig counting interface, and selects counting images by clicking the “IMAGE” button in the data collection section. The next step is to click the “DETECT-CPU” or “DETECT-GPU” button in the “Recognition Mode” section to recognize the input image. The recognition effect will be shown in the display part of the interface, and the counting result will be shown at “NUMBER:”.

As shown in Figure 16, when pig counting is completed, the number of pigs identified will be displayed in the system after the “NUMBER:” text box. This image contains a total of 29 pigs.

## 7. Conclusions

We propose a cross-scene pig identification and counting algorithm based on improved YOLOv5n. A cross-scenario pig counting dataset was created by selecting several pig datasets in different real scenarios, which enhances the generalization performance of the model in cross-scenarios. Using the FasterNet model as the Backbone of the YOLOv5n model reduces the number of parameters and computations and lays the foundation for the deployment of the model on mobile terminals. The Neck of the YOLOv5n was optimized by using the E-GFPN structure, which enhanced the fusion ability of the model and improved the influence of different occlusions, lighting, and imaging on the recognition effect of the model. The loss function of CIoU of YOLOv5n was replaced with the loss function of Focal EIoU, which further improved the model’s recognition accuracy of pigs.

The recognition effect of the proposed model in this paper was analyzed in different occlusion, different lighting, and different scenarios. Finally, the improved model was deployed on Android.

Results demonstrate that the AP of recognition by the proposed model is 97.72%, the number of parameters, the amount of calculation, and the size of the model are reduced by 50.57%, 32.20%, and 47.21% compared with the YOLOv5n model, and the speed of detection reaches 75.87 f/s. The mean absolute error of counts of the model in this paper is 1.37. The individual identification and counting algorithm proposed in this paper can improve the management of livestock and poultry farming and enhance the profitability of farming.

In future work, a combination of the algorithm proposed in this paper with the tracking algorithm can be used to realize real-time individual pig tracking and record the amount of activity so that abnormalities in activity can be detected and alerted quickly.

## Figures and Tables

**Table 1:** Pig counting dataset.

Dataset	Number of Images/Image	Number of Pigs/Head
Training	2236	54,596
Validation	186	4638
Testing	187	4714
Total	2609	63,948

**Table 2:** Hyperparameter setting.

Hyperparameter Name	Value
Image size	416 × 416 pixels
Batch size	32
Epoch	300
Num Workers	8
Initial Learning Rate	0.01
Momentum	0.937
Optimizer	SGD

**Table 3:** Detection accuracy of different lightweight models.

Backbone	Precision (%)	Recall (%)	F1-Score (%)	AP (%)
MobileNetv3small	94.28	85.47	89.66	93.34
ShuffleNetv2	95.33	84.48	89.58	93.65
PP-LCNet	95.31	85.98	90.40	94.28
FasterNet	94.72	89.42	91.99	95.64

**Table 4:** Comparison table of ablation experiment results.

Model	Improvement Points			AP (%)	Parameter (M)	Calculated Quantity (GFLOPs)	Model Size (MB)
	FasterNet	E-GFPN	Focal EIoU				
A	×	×	×	97.55	1.76	4.13	3.58
B	√	×	×	95.64	0.34	0.79	0.84
C	√	√	×	97.49	0.87	2.80	1.89
D	√	√	√	97.72	0.87	2.80	1.89

Note: √ indicates the use of this improvement point, × indicates the exclusion of this improvement point.

**Table 5:** Comparison table of the recognition results of different models.

Model	Precision (%)	Recall (%)	F1-Score (%)	AP (%)
YOLOv3-tiny	94.32	84.59	89.19	92.93
YOLOv4-tiny	95.01	87.57	91.14	94.60
YOLOv6n	96.58	95.18	95.87	97.45
YOLOv7-tiny	97.22	95.40	96.30	98.17
YOLOv5n	97.17	93.15	95.11	97.55
Our model	97.45	93.17	95.26	97.72

**Table 6:** Performance comparison table of different models.

Model	Parameter Quantity (M)	Calculated Quantity (GFLOPs)	Model Size (MB)	FPS (f/s)
YOLOv3-tiny	8.67	12.88	16.50	123.46
YOLOv4-tiny	3.06	6.30	5.92	120.19
YOLOv6n	4.30	4.67	9.29	42.79
YOLOv7-tiny	6.00	13.02	11.60	42.96
YOLOv5n	1.76	4.13	3.58	71.53
Our model	0.87	2.80	1.89	75.87

**Table 7:** Comparison of number of parameters and detection speed with existing methods.

Model	Parameter Quantity (M)	FPS (f/s)	Single Image Detection Time (s)
Tian et al. [1]	---	23.81	0.042
Yang et al. [3]	3.12	17.86	0.056
Ahn et al. [9]	52.92	33.51	0.030
Hu et al. [23]	86.06	2.50	0.400
Our model	0.87	75.87	0.013

**Table 8:** Comparison table of counting errors of each model.

Model	MAE	RMSE
YOLOv3-tiny	3.08	4.31
YOLOv4-tiny	2.77	4.10
YOLOv6n	1.46	2.18
YOLOv7-tiny	0.97	1.55
YOLOv5n	1.46	2.18
Our model	1.37	2.10

## Data Availability

Not applicable.
